# Draft Genome Sequence of the Multiple Antibiotic Producer Bacillus velezensis X-BIO-1

**DOI:** 10.1128/MRA.01319-20

**Published:** 2020-12-10

**Authors:** S. V. Kravchenko, D. V. Poshvina, A. V. Vasilchenko, A. S. Vasilchenko

**Affiliations:** a University of Tyumen, Institute of Environmental and Agricultural Biology (X-BIO), Laboratory of Antimicrobial Resistance, Tyumen, Russian Federation; b All-Russian Institute of Plant Protection, St. Petersburg-Pushkin, Russia; University of Rochester School of Medicine and Dentistry

## Abstract

Here, we describe the draft genome sequence of Bacillus velezensis strain X-BIO-1, which contains 16 contigs, comprising 3,861,135 bp with a G+C content of 46.54%. The annotated draft genome contains 3,710 protein-coding genes and 62 RNA genes. We identified genes responsible for the synthesis of various antibiotics.

## ANNOUNCEMENT

Bacillus velezensis is an aerobic, Gram-positive, endospore-forming, free-living soil bacterium with potential as a biopesticide against a broad spectrum of plant pathogens, including bacteria, fungi, and nematodes (1, 2).

Samples were collected from the soil of the Krasnodar region of Russia. B. velezensis strain X-BIO-1 was isolated as reported previously (3). The isolated strain was grown in Luria-Bertani (LB) broth at 37°C under aerobic conditions with shaking at 150 rpm for 24 h.

Genomic DNA was isolated from overnight broth culture using a routine method, which included mechanical homogenization followed by enzymatic lysis. Briefly, 300 µl of Tris-salt buffer (100 mM/liter Tris-HCl, 20 mM/liter EDTA, 750 mM/liter NaCl [pH 8.0]) was added to 50 µl sediment of the studied culture and homogenized using a TissueLyser LT instrument (Qiagen, Germany) with lyse matrix E (MP Biomedicals, USA) for 1 min at a frequency of 50 Hz. After that, a 10% SDS solution was added to the mixture to a final concentration of 1% with 4 µl of proteinase K solution (10 mg/ml) and incubated for 30 min at 60°C. After extraction with a mixture of phenol-chloroform-isoamyl alcohol (25:24:1) and subsequent extraction with chloroform-isoamyl alcohol (24:1), DNA was precipitated from the aqueous phase in 3 volumes of absolute ethanol with the addition of 10 M ammonium acetate (1:10) at −20°C overnight. After centrifugation and double washing with 80% ethanol, the DNA was dried and dissolved in 25 µl of deionized autoclaved water.

A DNA library was constructed using the NEBNext Ultra II FS library preparation kit (New England Biolabs, USA) according to the manufacturer's protocols. The size of the sequenced DNA fragment was 550 bp. Sequencing was performed with an Illumina MiSeq platform (Illumina, USA) in the Center of Shared Scientific Equipment “Microorganisms Persistence” of the Institute of Cellular and Intracellular Symbiosis of the Ural Branch of the Russian Academy of Sciences using a MiSeq reagent kit V3 with 2 × 300-bp reads.

As a result of sequencing, 1,550,219 paired-end reads were obtained. Default parameters were used for all software. Read quality analysis was performed using FastQC v0.11.5 software. Read filtration by quality (Q = 20) and length (removal of the first and last 10 nucleotides) was performed using Trimmomatic program v.0.39 (4) with the following parameters: LEADING:3 TRAILING:3 SLIDINGWINDOW:5:20 MINLEN:50 CROP:290 10. The genome was assembled using the Unicycler program (5), including assembly using the genome collector SPAdes v1.13.1 and polishing the contigs using Pilon v.1.23. The contigs resulting from the assembly were analyzed using the Quast program (6) to evaluate the assembly quality.

Prediction of genes encoding rRNA was performed using the Barrnap program (https://github.com/tseemann/barrnap). The whole-genome sequence of B. velezensis was annotated using the Prokaryotic Genome Annotation System (PROKKA) (7) and Rapid Annotation System Technology (RAST) server (8).

The resulting assembly of strain X-BIO-1 contained 16 contigs, comprising 3,861,135 bp. The *N*_50_ contig size was 978,250 bp, and the longest contig size was 1,102,097 bp. It had a G+C content of 46.54%, and average sequencing coverage was 192.54×. A total of 3,710 protein-coding genes were found in the assembly. The genome was shown to encode 2 rRNAs and 59 tRNAs. Protein-coding genes have been categorized into 322 different functional categories according to the SEED database, including identified genes responsible for the synthesis of aromatic compounds and antibiotics.

### Data availability.

The assembled genome sequence of B. velezensis strain X-BIO-1 was deposited in GenBank under accession no. JACBAX000000000.1. Illumina MiSeq raw reads have been deposited in the NCBI Sequence Read Archive under accession no. SRR12261816 (BioProject no. PRJNA642698, BioSample no. SAMN15396030).
